# IL-9 promotes the pathogenesis of ulcerative colitis through STAT3/SOCS3 signaling

**DOI:** 10.1042/BSR20181521

**Published:** 2018-11-28

**Authors:** Linglin Tian, Yuan Li, Jian Zhang, Ruqi Chang, Jianhong Li, Lijuan Huo

**Affiliations:** 1 Department of Gastroenterology, First Hospital of Shanxi Medical University, 85 Jie Fang South Road, Taiyuan 030001, Shanxi Province, People’s Republic of China; 2 First Clinical Medical College, Shanxi Medical University, 56 Xinjian South Road, Taiyuan, Shanxi 030001, People’s Republic of China

**Keywords:** IL-9, STAT3, SOCS3, Ulcerative colitis

## Abstract

Ulcerative colitis (UC) is a chronic condition in which the overreacting immune system may play an important role. It has been confirmed that the interleukin (IL) 9 (IL-9) participates in the pathogenesis of UC but the molecular mechanism is not fully illustrated. Here, we show that levels of peripheral blood cytokines IL-9, IL-8, IL-10, IL-6, IL-1β, IL-12, and tumor necrosis factor (TNF) were higher in patients with UC than normal control, and serum and local IL-9 levels were positively correlated with the disease activity grade. Moreover, IL-9 stimulation inhibited suppressor of cytokine signaling 3 (SOCS3) expression and wound healing ability in colonic epithelial cells and promoted the phosphorylation level of signal transducers and activators of transcription 3 (STAT3). And IL-9 stimulation promoted claudin-2 expression while inhibited claudin-3 and occludin expression. Furthermore, SOCS3 overexpression rescued the IL-9-induced effects. Altogether, IL-9 participates in the pathogenesis of UC through STAT3/SOCS3 signaling pathway and has the potential to serve as a possible therapeutic candidate in patients with UC.

## Introduction

Ulcerative colitis (UC) is characterized by chronic destructive, inflammatory disorders of the gastrointestinal tract. The cause of the overreacting immune response is unclear, but genetic, dietary, and environmental risk factors may play a role [1,2]. Patients with UC typically present with hematochezia, diarrhea, and abdominal pain. The onset of symptoms can be sudden or gradual [3]. The inflammation of UC is limited to the colonic mucosa and the portion of the colon affected varies: from the rectum (ulcerative proctitis) to UC that affects the entire colon [4]. Mucosal inflammation and ulcerations in UC are driven by activated cells of the immune system. And T lymphocytes produce large amounts of cytokines and induce tissue damage in patients with UC [5]. The pathogenesis of UC has partly uncovered in experimental animal models of the disease. Based on patient data and the murine oxazolone-induced colitis model, it has been demonstrated that epithelial barrier damage in UC is driven by a T-helper cell (Th) 2-like phenotype and interleukin (IL) 13 (IL-13) has been participating in disease pathogenesis [6,7].

Tissue integrity is important to the intestinal epithelium. Coordinate expression and interaction of proteins participate in the procession. The cytokines modulate the expression of tight junction proteins, regulate the assembly, and change the epithelial permeability dynamic nature of tight junctions [8]. In patients with UC and models of intestinal inflammation, this barrier gets compromised and harmful substances can enter the intestinal lumen and initiate disease [9,10]. IL-9 regulates barrier function through the regulation of claudin-2 in an experimental model of UC. And expression levels of occludin and claudin-3 were unaffected [11]. IL-9 first has been discovered as a potent growth factor for T cells and its role in allergic, asthmatic, and inflammatory conditions has been largely characterized recently [12,13]. As a Th2 cytokine, IL-9 has been recently identified to be the defining cytokine for a new lineage of CD4^+^ T cells, the Th9 cells, which additionally produce high levels of IL-9 [14,15]. The novel Th9 cells have been linked to the transcriptional programmers of signal transducer and activator of transcription (STAT) 6 (STAT6), interferon regulatory factor (IRF) 4, and PU.1 [16–18]. However, further studies are needed to explore the molecular mechanism underlying IL-9 roles in the pathogenesis of UC.

In the present study, we showed that the expression of human inflammatory cytokines including IL-9 was high and correlated with the disease grade in patients with UC compared with the normal control group. The molecular mechanism underlying IL-9 function in UC was further investigated. Our findings indicated that IL-9 promotes the pathogenesis of UC through STAT3/suppressor of cytokine signaling 3 (SOCS3) signaling pathway.

## Materials and methods

### Human tissues sample

Forty-four patients with UC were enrolled from January 2017 to January 2018. The diagnosis of UC was based on clinical history, endoscopic examination, and histopathological changes. The basic information of patients was obtained through consulting history information and on-the-spot inquiries. The normal control group was a healthy examination volunteer, without UC history, normal endoscopy, and no pathological examination abnormal. The UC active patients were divided into the mild, moderate, and severe grades. The project was approved by the Ethics Committee of First Hospital of Shanxi Medical University. The research has been carried out in accordance with the World Medical Association Declaration of Helsinki, and all subjects provided written informed consent.

### Cytometric bead array

Serum samples were spun at 3000 rpm for 10 min and the supernatant collected and tested in 2 h. The concentration of cytokines was measured using a cytometric bead array (CBA, BD Biosciences, CA, U.S.A.) as per manufacturer’s instructions (Human Inflammatory Cytokine Kit). CBA samples were run on FACS Calibur Flow Cytometer (BD Biosciences, CA, U.S.A.). Data were analyzed using FlowJo software (BD Biosciences, CA, U.S.A.) to convert fluorescent intensity values into concentrations using an 11-point standard curve (0–10000 pg/ml) and normalized to cell number.

### Cell lines and cell culture

RKO, Caco-2, NCM460, and SW480 cells were stored in our lab and cultured in 1640 medium (Invitrogen, Carlsbad, CA) supplemented with 10% FBS (Invitrogen, Carlsbad, CA), penicillin (100 U/ml), streptomycin (100 g/ml), nonessential amino acids (0.1 mM), and l-glutamine (2 mM) (Invitrogen, Carlsbad, CA).

### Immunohistochemistry in patients with UC and control subjects

Cryosections from colonic mucosa specimens of control subjects and patients with UC were used for immunohistochemistry as described. Tissues were fixed in 4% paraformaldehyde in PBS, followed by sequential incubation with avidin-biotin-blocking reagent (Vector Laboratories), peroxidase-blocking reagent, and protein-blocking reagent (Dako, U.S.A.) for suppression of nonspecific background staining. Sections were incubated with primary antibody to human IL-9 (Cell Signaling Technology, U.S.A.). Furthermore, some sections were incubated without primary antibody or with the appropriate isotype-matched control antibodies as negative controls. Subsequently, samples were incubated with the fluorescence–conjugated antibody (Dianova, CAN) or the secondary antibody biotinylated goat-anti-rabbit IgG or goat-anti-mouse IgG, followed by incubation with streptavidin–conjugated indocarbocyanine (Dianova, CAN) or staining with TSA-indocarbocyanine. Nuclei were counterstained with DAPI (Vector Laboratories) before final analysis by confocal microscopy (Leitz Microscope, U.S.A.). The intensity of IL-9 expression was graded as follows: negative = score 0, weak = score 1, moderated = score 2, and strong = score 3. Extent of staining was grouped according to the percentage of high-staining cells in the cancer nest: negative = score 0, 1–25% = score 1, 26–50% = score 2, 51–75% = score 3, and 76–100% = score 4. The final quantitation of each staining was obtained by multiplying the two scores. Low expression means refers to an IL-9 score ≤6, and high expression means refers to an IL-9 score >6. Immunoreactivity was assessed independently by two expert pathologists blind to all clinical data.

### Western blot analysis

Western blot was performed as follows. The dilution (1:1000) of primary antibodies as below: anti-GAPDH (Cell Signaling Technology), anti-STAT3 (Cell Signaling Technology), anti-pSTAT3 (Cell Signaling Technology), anti-SOCS3 (Cell Signaling Technology), claudin-2 (Abcam), Claudin-3 (Abcam), and occludin (Abcam), and cells were lysed in ice-cold RIPA buffer (50 mM Tris/HCl (pH 7.5), 150 mM NaCl, 1% Triton X-100, 0.1% SDS, and 0.5% sodium deoxycholate) supplemented with a protease inhibitor mixture (Sigma, St. Louis, MO). The lysates were kept on ice for 10 min, centrifuged, and resolved by SDS/PAGE (10% gel). The proteins were then transferred to a PVDF membrane (Pall, Port Washington, NY), blocked with 5% skim milk in PBST for 1 h, and probed with the indicated primary antibodies at an appropriate dilution overnight at 4°C. The following day, the membrane was incubated with the corresponding IRDye 800-labeled IgG secondary antibodies (Li-COR Inc., Lincoln, NE) and scanned using the Odyssey Infrared Imaging System (Li-COR Inc., Lincoln, NE).

### Real-time qRT-PCR analysis

Briefly, cells were cultured for RNA isolation using TRIzol reagent (Invitrogen, U.S.A.) following the manufacturer’s protocol. Then 1 μg total RNA was reverse transcribed to cDNA using random primers and first-strand cDNA synthesis SuperMix (TransGen Biotech, China). qRT-PCR was performed on a C1000 Thermal cycler (Bio-Rad, U.S.A.) with SYBR Green Mix (Takara Bio Inc, Japan). The primers were designed and synthesized by Sangon Biotech (Shanghai) Co., Ltd. All qRT-PCR products were verified by melting curve analysis. The real-time PCR primers were listed as follows: IL-9R forward: GTGGCCTTTCTTGTGACCAT, and reverse AGTCTCAGACAAGGGCTCCA. GAPDH forward: 5′-TGACTTCAACAGCGACACCCA-3′ and reverse: 5′- CACCCTGTTGCTGTAGCCAAA-3. The fold-relative enrichment was quantitated with normalization to the GAPDH level. Each experiment was repeated three times.

### Construction of recombinant lentiviral vector and transduction

The sequence of the *SOCS3* gene (NM_003955) was synthesized and cloned into the pGV492-SOCS3-GFP vector by the GeneChem Corporation. The pGV492-SOCS3-GFP, pHelper1.0, and pHelper2.0 were mixed and transfected into 293T cells according to the instructions of Lipofectamine 2000 (Invitrogen, Carlsbad, CA). Lentiviral particles were harvested from the cell after 48 h transfection, culture medium and then filtered through 0.45-μm PVDF membranes.

### Immunofluorescence assay

Cells were fixed in 4% formaldehyde, permeabilized in 0.5% Triton X-100, blocked in 5% BSA in PBS, and then probed with primary antibodies for 1 h at room temperature. Primary antibodies used were mouse anti-pSTAT3 (Sigma–Aldrich, St. Louis, MO). The cells were washed three times with PBS and then incubated with either goat anti-mouse Ig conjugated with Alexa Fluor 405 or goat anti-rabbit Ig conjugated with Alexa Fluor 594 at a dilution of 1:500 for 1 h (Invitrogen, Carlsbad, CA). The cells were then washed and stained with DAPI (Invitrogen, Carlsbad, CA) to detect nuclei. Fluorescence images were obtained and analyzed using an LSM 510 laser-scanning confocal microscope (Carl Zeiss, Germany).

### Wound healing assay

SW480, RKO cells were seeded into culture-inserts (Ibidi, Germany) placed on a cell culture surface according to manufacturer’s instructions and were grown to confluence. When confluence was reached the culture-inserts were removed leaving a cell-free gap, and cells were stimulated with 20 ng/ml IL-9 (Peprotech, U.S.A) for 60 min with or without 1 μg/ml αh-IL-9R (R&D Systems, U.S.A.) pretreatment (for 60 min, before IL-9 was added) or with culture medium alone. Pictures were taken at 0, 8, and 24 h after stimulation along the cell-free gap with an IX70 microscope (Olympus, Japan). ImageJ software was used to analyze the cell scratch area, wound healing rate = (scratch area in 0 h − a scratch area of different observation time)/scratch area in 0 h. The effect of the different stimulations was quantitated by measuring the distance between the approaching cell clusters in regular intervals.

### Statistical analysis

All data are expressed as means ± S.D. and were analyzed using SPSS 18.0 (Chicago, IL, U.S.A.). The statistical significance of the evaluated data was tested using the Student’s *t*test and Spearman’s rank correlation. The two groups were compared by the Mann–Whitney U test and Kruskal–Wallis test was used in multiple groups. Each bar represents the average of data points with the S.D. represented as the error bar. *P-*values <0.05 (*), <0.01 (**), or <0.001 (***) were considered significant for all analyses.

## Results

### Increased expression of IL-9 in the serum and colonic mucosa of patients with UC

**Figure 1:**
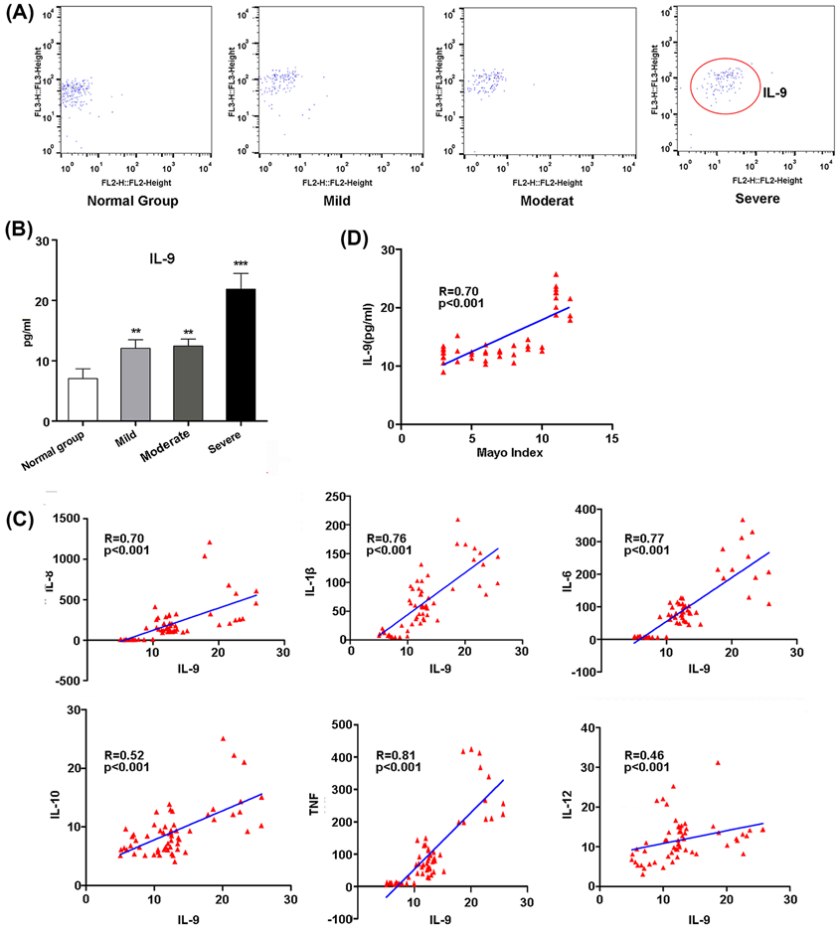
Expression of human inflammatory cytokines in serum from patients with UC (**A**) Expression of IL-9 in patients with different stages of UC and normal group by CBA. (**B**) The quantitative results of IL-9. The experiment was performed for three times, and data are shown as mean ± S.E.M. (**P<0.01; ***P<0.001). (**C**) Correlation of IL-9 level with levels of IL-8, IL-1β, IL-6, IL-10, TNF, and IL-12 in patients with UC and normal group. (**D**) Correlation of IL-9 level with the Mayo score of patients with UC.

To address the role of IL-9 in UC progress, we examined the IL-9 expression in 14 normal samples and 44 patients including 15 samples with mild active, 17 samples with moderate activity, and 12 samples with severe active expression. There was no significant difference in gender, age, treatment measures, and lesion range amongst different groups (Table 1). It has been confirmed that the *IL-9* mRNA in the colon mucosa of the patients with UC activity period is significantly higher than that of the normal population [7]. In this context, we first detected the IL-8, IL-1β, IL-6, IL-10, tumor necrosis factor (TNF), IL-12, and IL-9 levels in serum of different samples using CBA. The levels of IL-8, IL-1β, IL-6, IL-10, TNF, IL-12, and IL-9 in serum were gradually increased with the pathological progress of UC (Supplementary Figure S1 and Figure 1A,B). Besides, we also found that the expression of IL-9 was positively correlated with other inflammatory cytokines and Mayo Index (Figure 1C,D).

**Table 1:** Comparison of clinical data between the patients with UC and normal group

	Normal (*n*=14)	Mild (*n*=15)	Moderate (*n*=17)	Severe (*n*=12)
Gender				
Male	8	7	10	7
Female	6	8	7	5
Age (years)	44.3 ± 10.7	39.7 ± 12.5	42.5 ± 10.7	40.1 ± 11.3
Treatment measures				
Amino salicylic acid	-	15	17	12
Glucocorticoid	-	0	14	10
Immunosuppressant	-	0	0	2
Biologics	-	0	0	0
Lesion range				
E1	-	10	6	0
E2	-	5	7	4
E3	-	0	4	8
IL-9 low	14	10	6	2
IL-9 high	0	5	11	10

**Figure 2:**
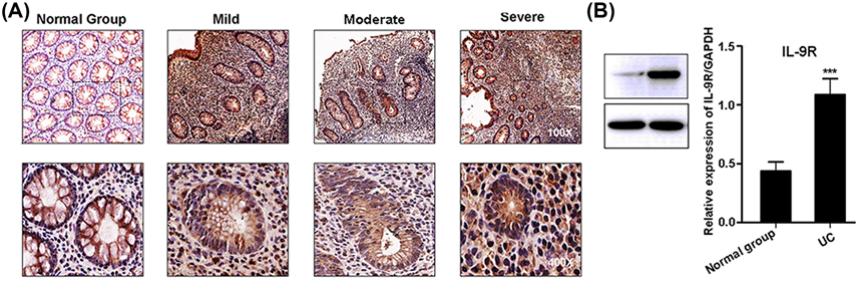
Patients with UC have higher expression of IL-9 and IL-9R (**A**) Expression of IL-9 in the colon mucosa of patients with different disease activity grades of UC and normal group was analyzed by immunohistochemistry. The IL-9 positive cells were only found in the cytoplasm of the colonic epithelial cells in the normal control group. And the expression of IL-9 in the inflammatory cells of the colon mucosa of the patients with UC activity grade was found and with enhanced staining. (**B**) Western blot was used to analyze IL-9R expression in patients with UC and normal group. And GAPDH was used for protein loading control. Each experiment was performed for three times, and data are shown as mean ± S.E.M. (***P<0.001).

To validate these results, we detected the expression of IL-9 in UC tissue using immunohistochemistry. As expected, the expression of IL-9 was positively correlated with the pathological progress of UC (Figure 2A and Table 1, χ^2^ = 22.086, *P*=0.00006). Furthermore, to identify potential targets of IL-9 signaling in patients with UC, the expression of IL-9R was detected in UC samples. It was found that the expression of IL-9R in the active UC patients was significantly increased compared with the normal group (Figure 2B). These results implied that the expression of IL-9 has a positive correlation with the progression of UC, and IL-9 may be a potential biomarker to predict the pathogenesis and progression of UC.

### IL-9 inhibits wound healing ability of colonic epithelial cells

**Figure 3:**
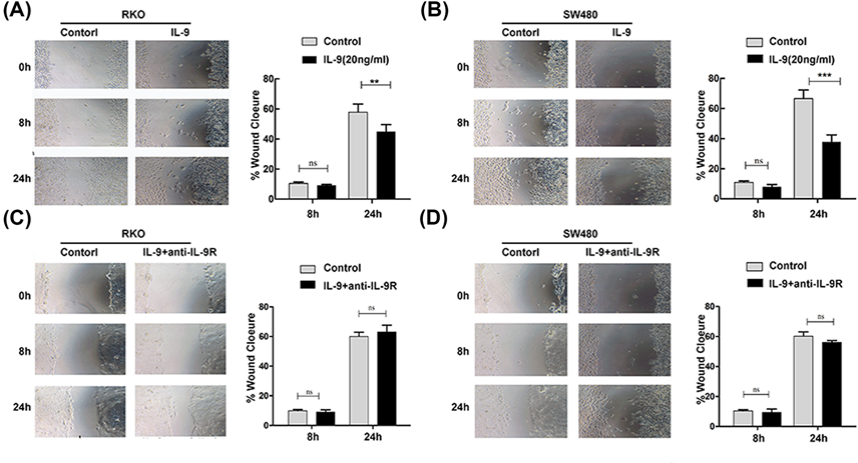
IL-9 inhibited wound healing by RKO and SW480 cell scratch assays (**A**,**B**) IL-9 (20 ng/ml) was added to RKO or SW480 cells grown to confluence on culture-inserts and cells were investigated in duplicates in three independent wound healing experiments. Each experiment was performed for three times, and data are shown as mean ± S.E.M. (**P<0.01; ***P<0.001; ns, no significance). (**C**,**D**) IL-9 (20 ng/ml), with or without anti-hIL-9R (1 μg/ml), was added to RKO or SW480 cells grown to confluence on culture-inserts and cell were investigated in duplicates in three independent wound healing experiments. Each experiment was performed for three times, and data are shown as mean ± S.E.M. (ns, no significance).

Wound healing experiment of the colonic epithelial cell was used to assess the gut physiology [19]. Due to the expression of IL-9R was higher in SW480 and RKO cells than Caco-2 and NCM460 cells (Supplementary Figure S2), we chose SW480 and RKO cells as a model for wound healing assay. As shown in Figure 3A,B, IL-9 significantly suppressed the wound healing ability of RKO and SW480 cells.

To clarify the role of IL-9 in cell wound healing ability, we blocked IL-9 receptor function using anti-IL-9R antibody. SW480 and RKO cells were treated by IL-9 after incubation of IL-9R receptor antibody (1 g/ml) for 1 h. The results showed that IL-9R blockade eliminated the effect of IL-9 on the growth of colonic epithelial cell (Figure 3C,D). These results indicated that IL-9 plays a potential pathogenic role in the context of mucosal healing in UC.

### IL-9 induces STAT3 phosphorylation in colonic epithelial cells

**Figure 4:**
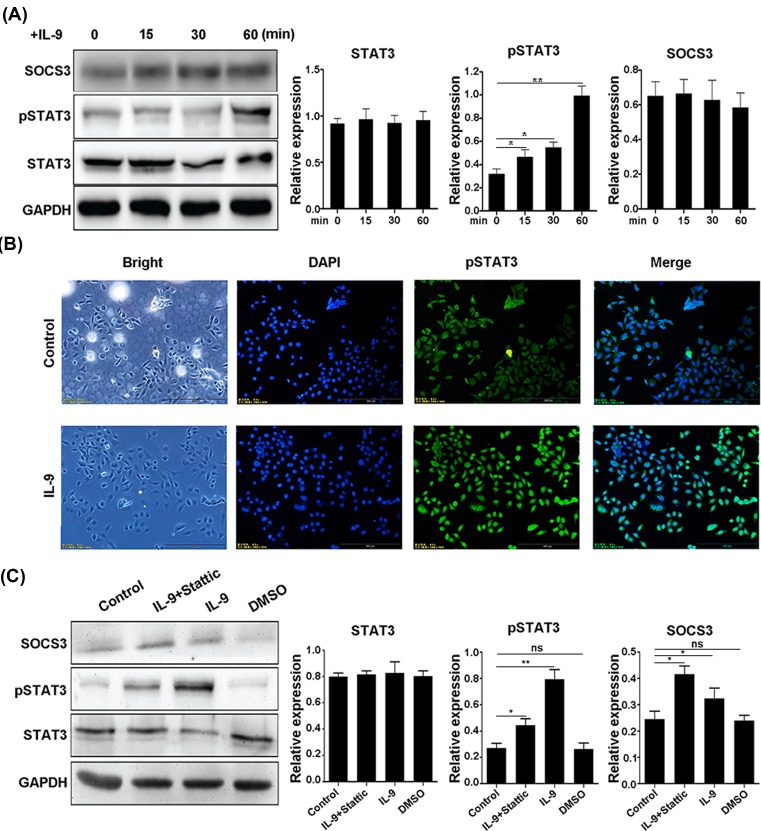
IL-9 induces STAT3 phosphorylation in SW480 cells (**A**) SW480 cells were stimulated with IL-9 and investigated for SOCS3, STAT3, and pSTAT3 expression (Left). Quantitation of SOCS3, STAT3, and pSTAT3 expression (Right). Each experiment was performed for three times, and data are shown as mean ± S.E.M. (*P<0.05; **P<0.01). (**B**) Immunofluorescence staining for pSTAT3 with or without IL-9 stimulation. (**C**) IL-9 (20 ng/ml), with or without anti-hIL-9R (1 μg/ml), was added to SW480 cells to verify the expression of SOCS3, STAT3, and pSTAT3 (Left). Quantitation of SOCS3, STAT3, and pSTAT3 expression (Right). Data are shown as mean ± S.E.M. Each experiment was performed three times, and data are shown as mean ± S.E.M. (*P<0.05; **P<0.01; ns, no significance).

It has been reported that IL-9 may affect inflammation through STAT3 signaling pathway [15]. Next, we detected whether IL-9 inhibited wound healing ability of colonic epithelial cells via STAT3 signaling pathway. In SW480 cells, Western blotting analysis showed that the IL-9 could increase the phosphorylation of STAT3, while the total protein of STAT3 was not changed (Figure 4A), and the similar result was observed in the immunofluorescence assay (Figure 4B). On the contrary, the expression of SOCS3 was inhibited after IL-9 stimulation (Figure 4A).

To further validate the effect of IL-9 on the pSTAT3 level in SW480 cells, the stattic (STAT3 activation inhibitor) was used to inhibit the STAT3 activation in SW480 cells. As expected, stattic partly suppressed the phosphorylation of STAT3, while the expression level of SOCS3 protein in SW480 cells increased in IL-9 and static stimulation group compared with IL-9 incubation group alone, suggesting that IL-9 may regulate SOCS3 expression through the pathway of STAT3 (Figure 4C).

### IL-9 affects expression of intestinal barrier related in colonic epithelial cells

**Figure 5:**
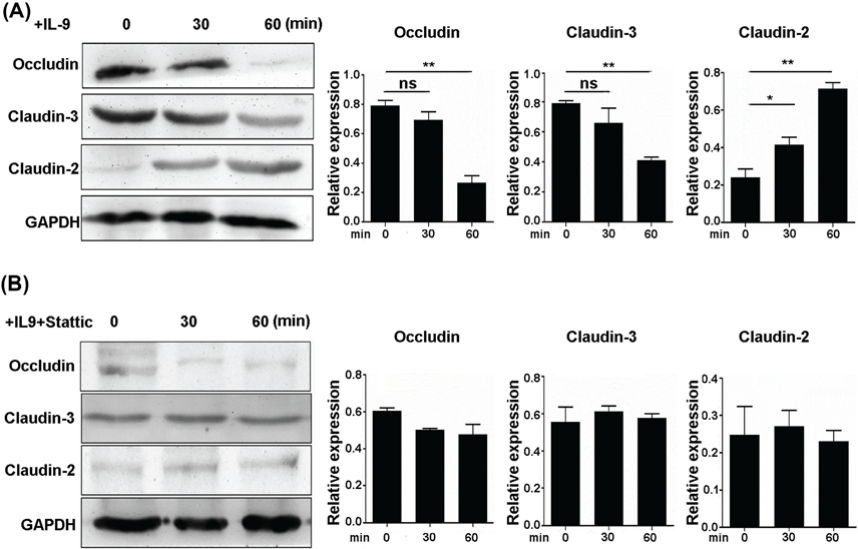
Effects of IL-9 on intestinal barrier related gene expression in colonic epithelial cells (**A**) SW480 cells were stimulated with 20 ng/ml IL-9 and investigated for occludin, claudin-3, and claudin-2 expression (Left). Quantitation of occludin, claudin-3, and claudin-2 expression (Right). GAPDH was used as a control. Each experiment was performed for three times, and data are shown as mean ± S.E.M. (*P<0.05; **P<0.01; ns, no significance). (**B**) IL-9 (20 ng/ml), with or without anti-hIL-9R (1 μg/ml), was added to SW480 cells to verify the expression of occludin, claudin-3, and claudin-2 (Left). Quantitation of occludin, claudin-3, and claudin-2 expression (Right). GAPDH was used as a control. Each experiment was performed for three times, and data are shown as mean ± S.E.M.

Next, we evaluated the effect of IL-9 on the expression of intestinal barrier related gene. After IL-9 stimulation, expression of claudin-2 was increased while the expression of claudin-3 and occludin were declined (Figure 5A). Interestingly, inhibiting the STAT3 activation by static reversed the expression of intestinal barrier related gene changing (Figure 5B). These findings suggested that IL-9 could disrupt the intestinal mucosal barrier through STAT3 signaling.

### SOCS3 overexpression rescues IL-9-induced STAT3 phosphorylation and IL-9/STAT3-mediated colonic mucosal injury

**Figure 6:**
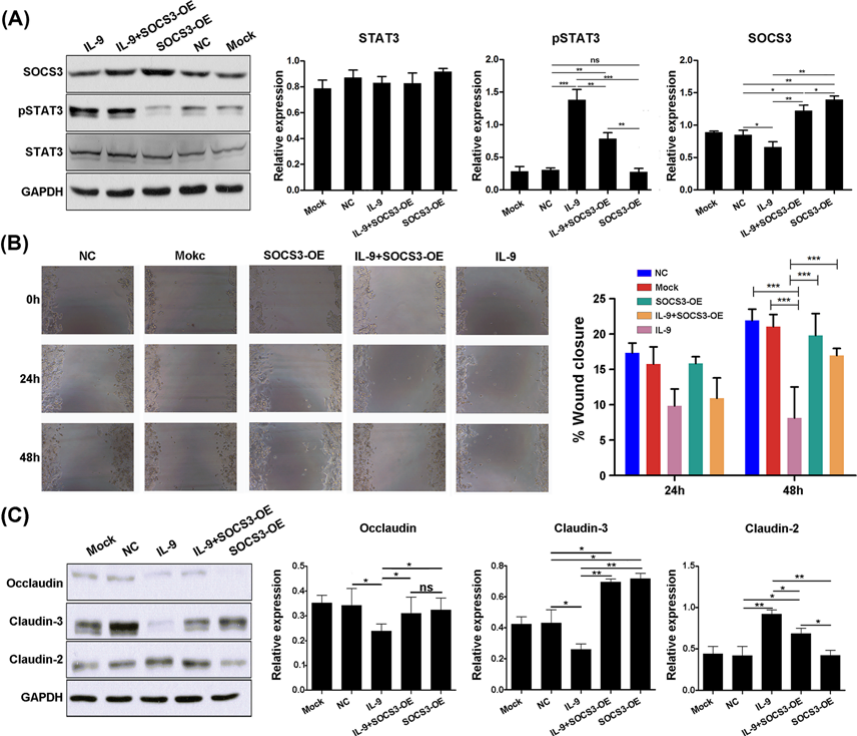
Overexpression of SOCS3 inhibits IL-9/STAT3-mediated colonic mucosal injury and effect to intestinal barrier related gene expression (**A**) SW480 cells were pretreated with SOCS3 overexpression lentivirus (OE), with or without IL-9 stimulation 24 h later. Expression of occludin, claudin-3, and claudin-2 were extracted for analysis by Western blot (Left). SOCS3, STAT3, and pSTAT3 expression were quantitated (Right). GAPDH was used as a control. Each experiment was performed for three times, and data are shown as mean ± S.E.M. (*P<0.05; **P<0.01; ***P<0.001; ns, no significance). (**B**) Wound healing ability of SW480 cell was assayed by scratch assays; 24 and 48 h after IL-9 stimulation, cell confluence was pictured. Representative pictures of one experiment are shown (Left) and quantitation of lesion size as indicated (Right). Each experiment was performed for three times, and data are shown as mean ± S.E.M. (***P<0.001). (**C**) SW480 cells were pretreated with SOCS3 overexpression lentivirus (OE), with or without IL-9 stimulation 24 h later. Expression of occludin, claudin-3, and claudin-2 were extracted for analysis by Western blot (Left). Occludin, Claudin-3, and claudin-2 expression were quantitated (Right). GAPDH was used as a control. Each experiment was performed for three times, and data are shown as mean ± S.E.M. (*P<0.05; **P<0.01; ns, no significance).

Above results showed that IL-9 could inhibit the SOCS3 expression and induced the injury of colonic mucosa cells. To validate this conclusion, the rescue experiment was designed to detect the role of SOCS3 in the IL-9/STAT3-induced injury of colonic mucosa cells. After lentivirus infection, SOCS3 was significantly overexpressed (Supplementary Figure S3). The result showed that SOCS3 overexpression partly rescued the phosphorylation of STAT3 (Figure 6A). Next, cell scratch assay was used to access the effect of SOCS3 overexpression on IL-9/STAT3-mediated colonic mucosal injury. The results showed that compared with group NC and mock group, the wound healing rate of SW480 cell was decreased 48 h after IL-9 incubation. While, compared with the simple IL-9 incubation, the wound healing rate in the SOCS3 overexpression combined with the IL-9 group was significantly increased, suggesting that SOCS3 overexpression could improve the decrease in IL-9-induced cellular wound healing ability of colon epithelial cells (Figure 6B).

Effects of SOCS3 overexpression on intestinal barrier related gene expression in colonic epithelial cells were accessed. As showed in Figure 6C, the results suggested that SOCS3 overexpression could inhibit the IL-9 stimulation induced claudin-2 enhancement. Furthermore, SOCS3 overexpression could up-regulate claudin-3 expression (Figure 6C). Taken together, these results suggest that SOCS3 overexpression inhibits IL-9-induced claudin-2 expression and up-regulates claudin-3 expression, thus improving intestinal barrier.

## Discussion

In the present study, by investigating pro-inflammatory factors IL-8, IL-6, IL-1β, IL-10, TNF, IL-12, and IL-9 level in serum in patients with UC, we found that they correlated with the grade of UC. These findings were consistent with previous research that cytokines including IL-8 could serve as prediction therapeutic biomarkers in patients with UC [19]. The altered expression of IL-6 reportedly participated in the pathogenesis of UC [20]. Moreover, it was reported that an elevation of circulating IL-9 in patients with inflammatory bowel disease and correlate to a severe prognosis [21]. Given the important role in the pathogenesis of UC, IL-9 was chosen for further study. To extend the effect of IL-9 *in situ*, we stained paraffin gut biopsies and resected tissues from patients with UC with the mild, moderate, and severe group as well as a normal group. We found the expression of IL-9 and IL-9R expression in patients with UC was significantly increased.

The role of IL-9 in the process of mucosal healing is not fully understood, we evaluated its effect on intestinal epithelial cells. IL-9 significantly inhibited the growth of RKO and SW480 cells into the gap in scratch assays, which was consistent with the report that systemic IL-9 level corresponds with endoscopic inflammation, may serve as a negative marker of mucosal healing in UC [22,23]. Moreover, SW480 cells were stimulated freshly with IL-9, the level of pSTAT3 increased but SOCS3 did not change. SOCS family are induced by a wide range of cytokines, including IL-1, IL-3, IL-4, IL-6, erythropoietin, IFNγ, leukemia inhibitory factor (LIF), granulocyte colony-stimulating factor, granulocyte macrophage colony-stimulating factor, and growth hormone (GH) [24]. SOCS3 is a repressor of the STAT3 signaling pathway. In addition, SOCS3 plays a negative feedback regulator of STAT3 [25]. Immunofluorescence staining results suggest that pSTAT3 translocated from cytoplasm to nucleus and participated in signal transduction. Rescue experiment using STAT3 activation inhibitor shown that IL-9 regulates SOCS3 expression through the pathway of STAT3.

The expression of primary sealing and integral membrane components of the tight junction in patients with UC are not yet fully characterized. Recently, occludin and the family of claudin proteins are well-defined junctional molecules [26]. Here, we found that claudin-2 was increased after IL-9 incubation, and the expression of claudin-3 and occludin were declined, respectively. The altered barrier function in the gastrointestinal tract during inflammation leads to loss of solutes and results in diarrhea [9]. A differential expression pattern of occludin, claudin-2, and claudin-3 induced by IL-9 in colonic inflammation may open up a new perspective in the pathogenesis of this disease, which warrants further investigation. Moreover, SOCS3 overexpression inhibits IL-9 induced claudin-2 expression and up-regulate claudin-3 expression and further improve intestinal barrier. Taken together, STAT3/SOCS3 signaling pathway may be affected by IL-9 stimulation.

In summary, our study demonstrates that IL-9 participates in the pathogenesis of UC by inducing signaling pathway of STAT3. Therefore, IL-9 could serve as a possible severity marker and a promising therapeutic candidate in patients with UC.

## Supporting information

**Supplemental figure 1:** Expression of human inflammatory cytokines in serum from patients with UC.

**Supplemental figure 2:** The relative expression of IL-9R in four cell lines

**Supplemental figure 3:** Detection of SOCS3 overexpression by Western 11 blot in SW480 cells.

## Abbreviations

CBA: cytometric bead array
IL: interleukin
SOCS3: suppressor of cytokine signaling 3
STAT: signal transducer and activator of transcription
Th: T-helper cell
TNF: tumor necrosis factor
UC: ulcerative colitis
