# Supplementary material for: IL-9 promotes the pathogenesis of ulcerative colitis through STAT3/SOCS3 signaling
Source: Biosci Rep. 2018 Nov 28;38(6):BSR20181521. doi: 10.1042/BSR20181521 (PMC6259019; doi:10.1042/BSR20181521)
Supplement: Supplementary file 1 [file bsr20181521_Supp1.pdf]

Supplemental information

Supplemental figure 1. Expression of human inflammatory cytokines in serum from patients with UC.

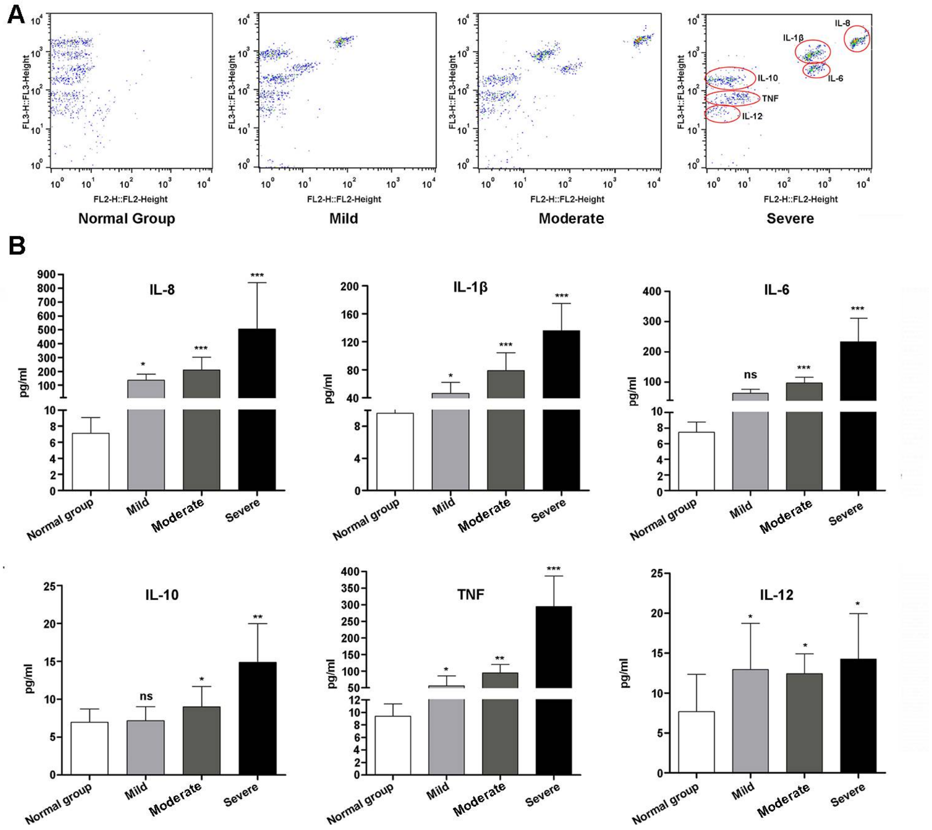

(A) Expression of IL-8, IL-1β, IL-6, IL-10, TNF and IL-12 in patients with different stages of UC and normal group by cytometric bead array.

(B) The quantitative results of IL-8, IL-1β, IL-6, IL-10, TNF and IL-12. Data are shown as mean values ± SEM (*p<0.05, **p<0.01, ***p<0.001).

Supplemental figure 2. The relative expression of IL-9R in four cell lines

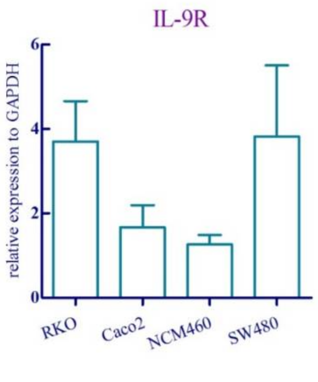

Expression of IL-9R mRNA was measured by q-PCR in four cell lines, RKO, Caco2, NCM460 and SW480.

Supplemental figure 3. Detection of SOCS3 overexpression by Western blot in SW480 cells.

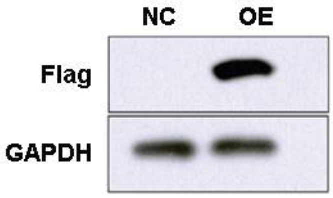

Expression of SOCS3 protein was measured by western blot in SW480 cell. GAPDH was used as internal control.
